# Knockdown of NUPR1 inhibits angiogenesis in lung cancer through IRE1/XBP1 and PERK/eIF2α/ATF4 signaling pathways

**DOI:** 10.1515/med-2023-0796

**Published:** 2023-10-16

**Authors:** Lihuai Wang, Jing Wen, Yinhui Sun, Xiao Yang, Yi Ma, Xuefei Tian

**Affiliations:** School of Integrated Chinese and Western Medicine, Hunan University of Chinese Medicine, Changsha, Hunan, 410208, China; Department of Oncology, The First Affiliated Hospital of Hunan University of Chinese Medicine, Changsha, Hunan, 410000, China; School of Medicine, Hunan University of Chinese Medicine, Changsha, Hunan, 410208, China; College of Integrated Chinese and Western Medicine, Hunan University of Chinese Medicine, No. 300 Xueshi Road, Yuelu District, Changsha, Hunan, 410208, China

**Keywords:** NUPR1, lung cancer, angiogenesis, signaling pathways

## Abstract

The stress response molecule nuclear protein‑1 (NUPR1) is essential for the growth of multiple types of human malignant tumor cells. However, the significance of NUPR1 in lung cancer is still not entirely elucidated. Therefore, this study is aimed to explore the function and underlying mechanisms of NUPR1 in lung cancer. NUPR1 mRNA and protein levels in lung cancer cell lines (A549 or H1299 cells) were silenced through siRNA transfection and western blot observed its successful infection efficiency. Then, using tube formation and wound healing experiments, the effects of si-NUPR1 on angiogenesis and migration of human umbilical vein endothelial cells (HUVEC) were examined, respectively, which indicated inhibitory effects on the angiogenesis and migration of HUVEC. Vascular endothelial growth factor A (VEGFA), a vital molecule in angiogenesis, was detected by PCR and western blot assays, manifesting NUPR1 knockdown represses VEGFA expression. Furthermore, the knockdown of NUPR1 may reduce angiogenesis by lowering VEGFA expression through inositol-requiring enzyme 1 (IRE1)/X box binding protein 1 (XBP1) and protein kinase RNA-like endoplasmic reticulum kinase (PERK)/eukaryotic translation initiation factor 2 A (eIF2α)/recombinant activating transcription factor 4 (ATF4) signaling pathways in A549 or H1299 cells. In conclusion, these findings demonstrated that NUPR1 knockdown inhibits angiogenesis in A549 and H1299 cells through IRE1/XBP1 and PERK/eIF2α/ATF4 signaling pathways, indicating that NUPR1 could represent a novel lung cancer therapeutic target.

## 1 Introduction

Lung cancer is among the most prevalent and lethal forms of cancer, having a high mortality rate [1]. Lung cancer is usually characterized by early migration and invasion, with richly vascularized tumor tissues [1]. Non-small cell lung cancer (NSCLC), which comprises adenocarcinoma, large cell carcinoma, and squamous carcinoma, is the predominant subtype of lung cancer, comprising approximately 80% of lung malignancies [1,2]. Despite significant breakthroughs in lung cancer treatment, such as the development of new medications and treatment alternatives, patients with NSCLC continue to have a dismal 5-year survival rate [3]. Consequently, it is essential to investigate the molecular processes underlying the advancement of NSCLC.

Endoplasmic reticulum (ER) buildup of misfolded or unfolded proteins is a characteristic of all malignancies [4], including NSCLC [5]. Around 55.9–87.5% of NSCLC tissues are under ER stress [5]. Efficient reduction of such protein folding load is required to maintain ER homeostasis, and the reduction impact is predominantly through the unfolded protein response (UPR), an evolutionarily conserved signaling pathway in eukaryotic cells [5,6]. Interestingly, UPR activation is required for high proliferation and chemoresistance in NSCLC cells [5].

Angiogenesis, characterized by the germination, migration, and remodeling of existing blood vessels, is crucial to the development of NSCLC, and vascular endothelial growth factor A (VEGFA) involves in the progress of angiogenesis [7]. Furthermore, activated UPR has been found to result in the upregulation of many pro-angiogenic factors (such as VEGFA) through inducing transcription factors inositol-requiring enzyme 1 (XBP-1) and activating transcription factor 4 (ATF4) to contribute to stabilizing VEGFA mRNA and stimulating VEGFA transcription [8].

Nuclear protein 1 (NUPR1), a stress-induced protein, has been found to represent an intriguing link between cellular and ER stress, which plays an essential part in the development of a number of malignancies, including pancreatic, breast, and prostate cancers [9]. In addition, NUPR1 has been reported to contribute to the progression of lung cancer [10]. Our previous study also confirmed that the knockdown of NUPR1 inhibited UPR-related factors (including UPR, XBP1, and ATF4) [11]. It has been shown that NUPR1 enhances VEGFA-mediated angiogenesis in hepatocellular carcinoma [12]. Unfortunately, the involvement of NUPR1 in lung cancer angiogenesis and related mechanisms are unclear.

In this study, the effect of NUPR1 in lung cancer cell lines was estimated. It was found that NUPR1 silencing could inhibit angiogenesis in human umbilical vein endothelial cells (HUVEC) cells, and these inhibitory effects may exert through reducing VEGFA expression and modulating UPR-induced transcription factors (inositol-requiring enzyme 1 [IRE1]/XBP1 and protein kinase RNA-like endoplasmic reticulum kinase [PERK]/eukaryotic translation initiation factor 2 A [eIF2α]/ATF4).

## 2 Methods

### 2.1 Cell culture in hypoxic

American Type Culture Collection was the source for the A549 (CRM-CCL-185) and H1299 (CRL-5809) human lung cancer cell lines. They were cultivated in RPMI-1640 media (Sigma Aldrich, MFCD00217820) with 10% fetal bovine serum (FBS) (Gibco, 10099-141) under a hypoxic environment (5% CO_2_, 1% O_2_, and 94% N_2_).

### 2.2 Downregulation of NUPR1 gene by siRNA

The NUPR1 gene was knockdown using the siRNA transfection kit (Shanghai GeneChem Co., Ltd, China): NUPR1 siRNA #1 5ʹ-GGAUGAAUCUGACCUCUAUTT-3ʹ and NUPR1 siRNA #2 5ʹ-GGCAGCAACAAUAAAUAGATT-3ʹ. By adding Lipofectamine RNAiMAX complex (Invitrogen, Carlsbad, CA, USA) containing siRNA to the culture media, siRNA transfection of A549 and H1299 cells was initiated. A well contained Lipofectamine RNAiMAX and siRNA and the ratio was 1.5 μL:5 pmol per well. Silencer Select Negative Control #1 siRNA (Ambion) was employed as a negative control (NC) for the siRNA therapy.

### 2.3 Tube formation assay

Growth factor-reduced Matrigel Basement Membrane Matrix (BD Biosciences, USA) was thawed gently on ice. A 96-well plate was then added with 50 μL of polymerization solution. Lung cancer cells were cultivated under hypoxic environment (5% CO_2_, 1% O_2_, and 94% N_2_) and incubated for 24 h on a total of 1 × 10^4^ HUVECs plated on the top of the Matrigel matrix. Finally, the capillary network was examined by counting all the tubes in ten different microscopic regions at random.

### 2.4 Wound healing

In six-well plates, the A549 and H1299 cells were cultivated till confluence. After treatments, confluent monolayers were scraped to form a linear wound by starving the cells for an overnight period in a medium containing 1% FBS (Gibco). The cells were washed and then cultivated for 24 h in complete media. The wounds were captured on camera using a phase contrast microscope.

### 2.5 Reverse transcription quantitative PCR

Using the 2^−ΔΔCT^ method, the mRNA relative expression level was quantified. Total RNA was extracted and then its concentration and purity were determined through a spectrophotometer (Thermo Fisher Scientific, Inc., MA, USA). Subsequently, using the StepOne™ PCR amplifier (Applied Biosystems, USA) and SYBR Premix Ex Taq™ II PCR kit (Takara Biotechnology Co., Ltd) following the manufacturer’s instructions, appropriate primer-coated RNA was reverse transcribed into cDNA. The primer sequences of NUPR1 were 5ʹ-GGAAAGGTCGCACCAAGAGAG-3ʹ (forward) and 5ʹ-ACCAGTTTCCTCTCGTGCCC-3ʹ (reverse). The primer sequences of β-actin, as a control group, were 5ʹ-CATGTACGTTGCTATCCAGGC-3ʹ (forward) and 5ʹ-CTCCTTAATGTCACGCACGAT-3ʹ (reverse).

### 2.6 Western blot

By using western blot, NUPR1, VEGFA, PERK, p-eIF2α, eIF2α, ATF4, IRE1, XBP1, and β-actin expression in A549 and H1299 cells were assessed. The protein debris was removed by centrifuging (Thermo Fisher, 75004061) at 12,000 rpm for 10 min at 4°C, after extraction with RIPA buffer (Beyotime, Shanghai, China). Protein concentration was then quantified using a bicinchoninic acid kit (Beyotime, Shanghai, China). Equivalent quantities (10 μg) of denaturized proteins were electrophoretically separated based on the molecular weight before being transferred to polyvinylidene difluoride membranes (LMAI Bio, LM1136). The membranes were blocked with 5% nonfat dry milk at room temperature for 1 h. The membrane was then treated with primary antibodies at 4°C overnight, rinsed with tris-buffered saline with 0.1% Tween^®^ 20 detergent, and incubated with corresponding secondary antibodies at 37°C for 1 h. The emitter coupled logic detection technique was then used to identify particular protein bands in membranes (GE Healthcare, Piscataway, NJ, USA).

Antibodies against NUPR1 (#ab234696, 1:1,000), VEGFA (#ab52917, 1:1,000), PERK (#ab229912, 1:1,000), eIF2α (#ab169528, 1:1,000), ATF4 (#ab270980, 1:1,000), IRE1 (#ab124945, 1:1,000), XBP1 (#ab220783, 1:1,000), and β-actin (#ab8226, 1:1,000) were purchased from Abcam. Anti-mouse (#4410, 1:10,000) and anti-rabbit (#4414, 1:10,000) peroxidase-conjugated secondary antibodies were acquired from Cell Signaling Technologies.

### 2.7 Statistical analysis

Using SPSS 22.0 (IBM, Armonk, NY, USA), all data were statistically evaluated. Student’s *t*-test was utilized to identify significant differences. The findings of three separate trials are shown as the mean ± standard deviation (SD). *p*  ≤  0.05 was considered to be statistically significant.

## 3 Results

### 3.1 NUPR1 knockdown inhibits angiogenesis in lung cancer cells

**Figure 1:**
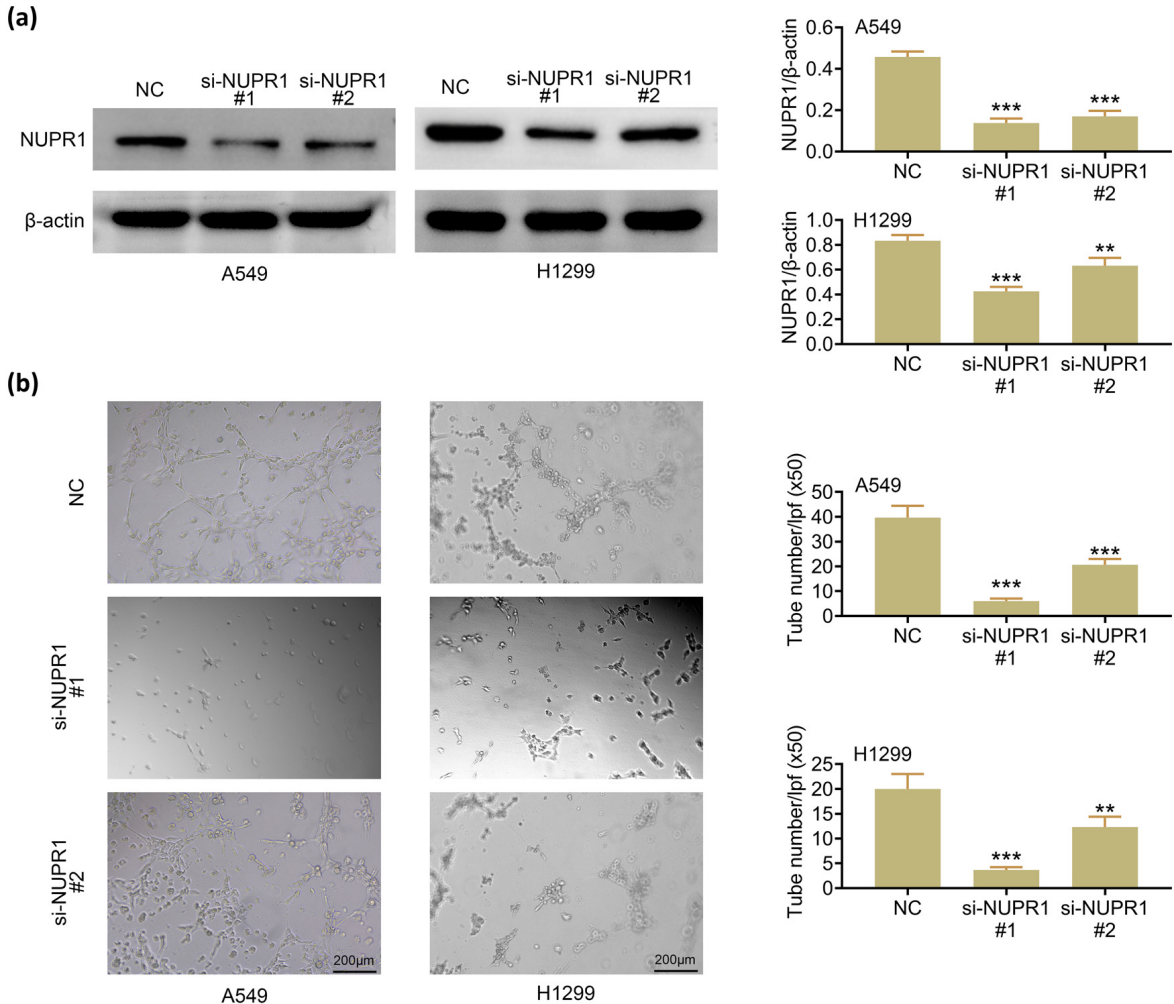
NUPR1 knockdown inhibits angiogenesis in lung cancer cells: (a) expression of NUPR1 was detected by western blot and normalized with β-actin and (b) tube formation assays were performed to measure angiogenesis in HUVEC cells, which were cultured in si-NUPR1-transfected lung cancer cells medium. Data represent the mean ± SD. **p < 0.01, ***p < 0.001 vs the NC group.

To better understand the significance of NUPR1 in the lung cancer cell lines, the siRNA against NUPR1 was used for gene silencing in the A549 and H1299 cells. The expression protein levels of NUPR1 were detected using western blot, the results of which revealed that NUPR1 protein expression has a significant reduction in the si-NUPR1#1 and i-NUPR1#2 groups compared to that in the NC group (Figure 1a).

Angiogenesis is critical for tumorigenesis, migration, and invasion and the hypoxic microenvironment of tumors may further induce angiogenesis [3]. Moreover, NUPR1 has been reported to have an angiogenic effect [12]. Then, in this study, the supernatants of si-NUPR1-transfected cells were obtained to analyze HUVEC cell tube development. The accumulated number of tubes in the si-NUPR1#1 and si-NUPR1#2 supernatant groups was much lower than in the sh-NC group (Figure 1b). Together, our findings suggested that inhibiting NUPR1 expression might reduce angiogenesis in lung cancer.

### 3.2 NUPR1 knockdown decreases cell migration of HUVEC cells

**Figure 2:**
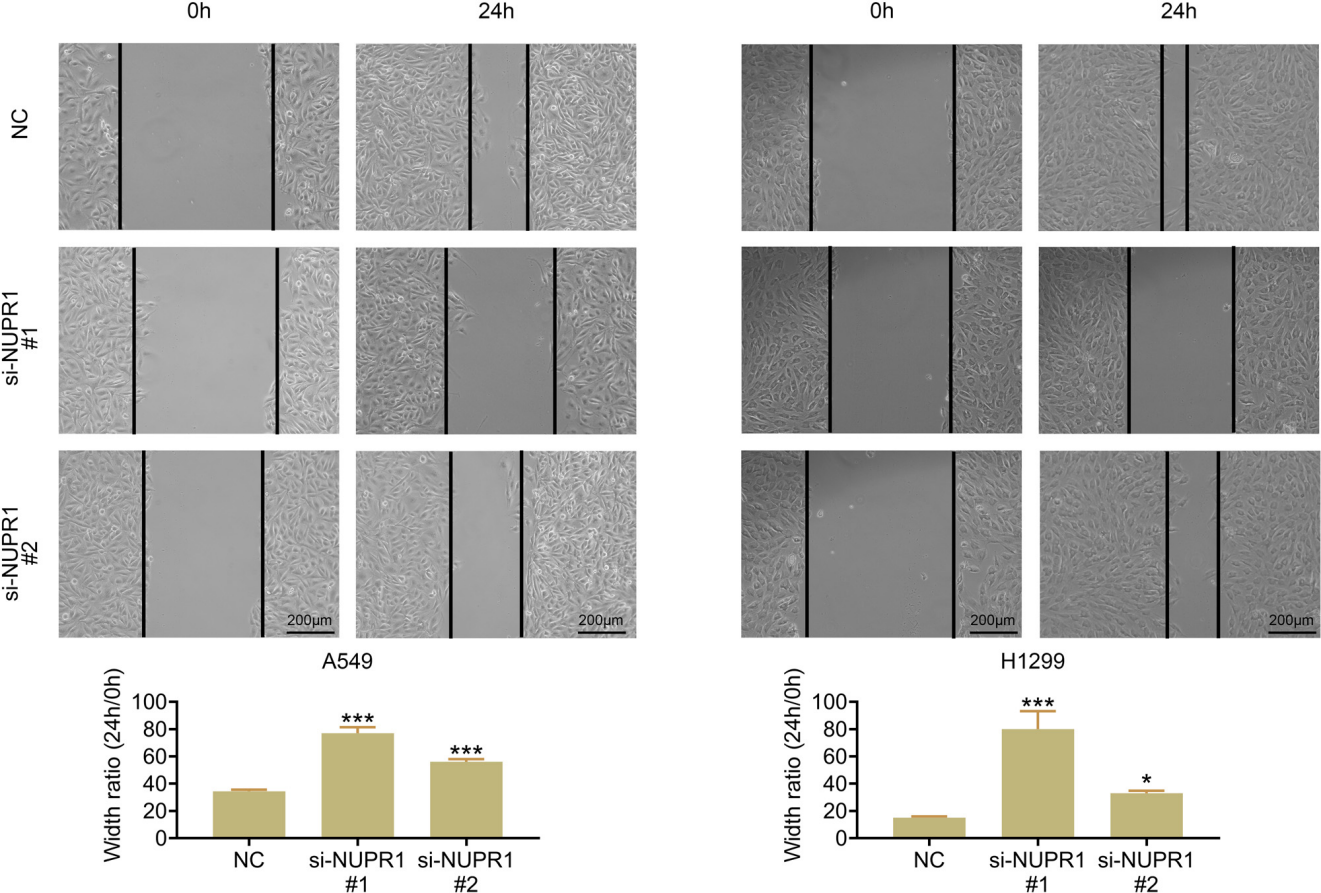
NUPR1 knockdown decreases cell migration of HUVEC cells. Wound healing analysis was performed to measure cell migration. Scale bar, 100 μm. Data represent the mean ± SD. *p < 0.05, ***p < 0.001 vs the NC group.

Some studies showed that HUVEC cell migration is essential for tumor angiogenesis, accelerating tumor progression [3]. Hence, a wound-healing experiment was conducted to determine the effect of NUPR1 on HUVEC cells. HUVEC cells were incubated with medium from A549 and H1299 cells, which were cultivated under hypoxic environments. The analysis confirmed that a medium with NUPR1 deletion could markedly reduce the number of migrative HUVEC cells (Figure 2). Collectively, the result suggested that NUPR1 knockdown could repress HUEVC cell migration.

### 3.3 NUPR1 knockdown suppresses VEGFA expression

**Figure 3:**
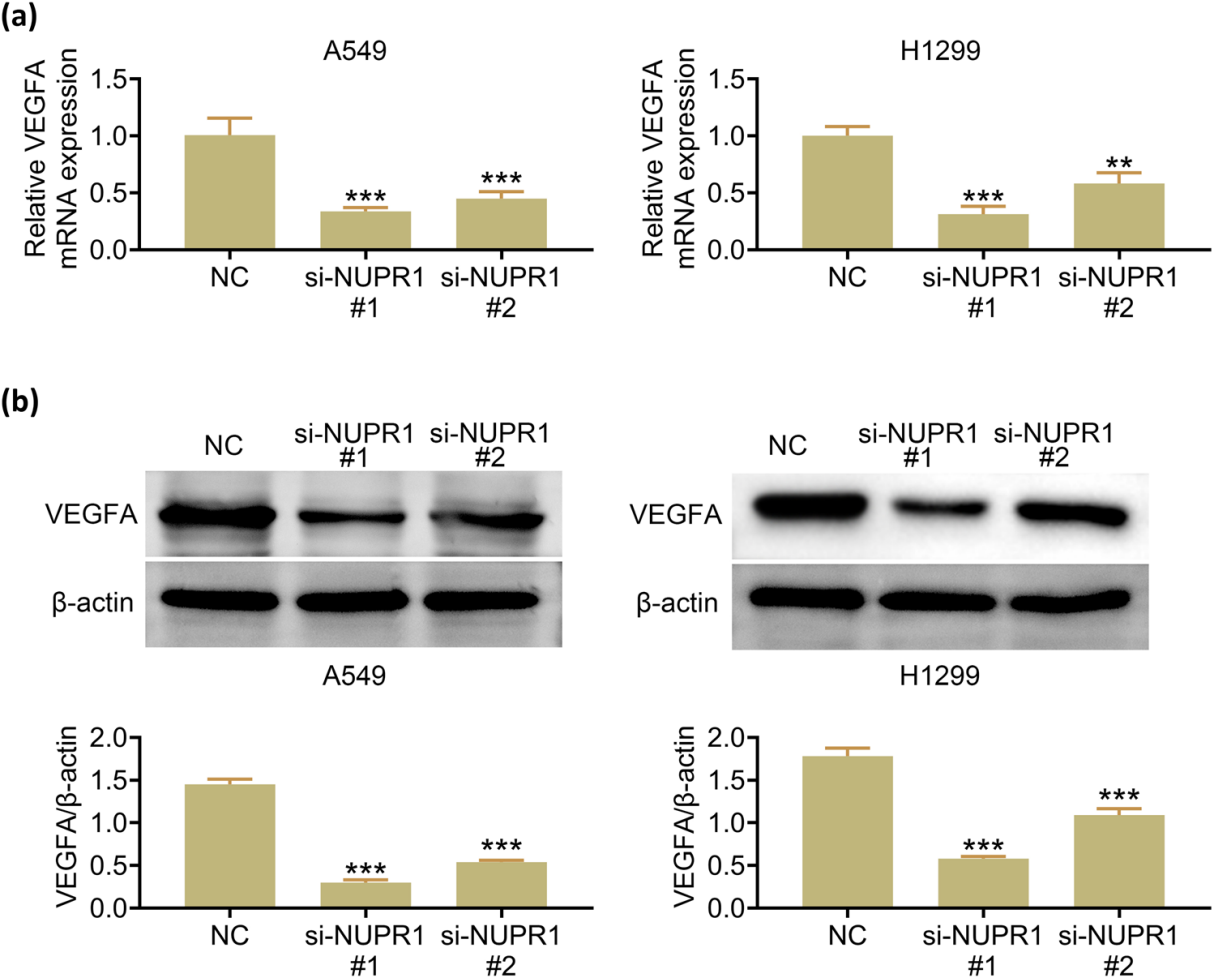
NUPR1 knockdown suppresses VEGFA expression: (a) RT-qPCR analysis of VEGFA mRNA levels and (b) western blot analysis of VEGFA protein expression levels. Data represent the mean ± SD. **p < 0.01, ***p < 0.001 vs the NC group.

VEGFA is pivotal in controlling angiogenesis, and the hypoxic status in tumor tissues could upregulate the secretion of the pro-angiogenic factor VEGFA [13]. Thus, the mRNA and protein expression levels of VEGFA were assessed by PCR and western blot, respectively. These findings showed the knockdown of NUPR1 in A549 and H1299 cells markedly decreased the expression levels of VEGFA (Figure 3a and b).

### 3.4 NUPR1 knockdown represses VEGFA expression through XBP1 and ATF4

**Figure 4:**
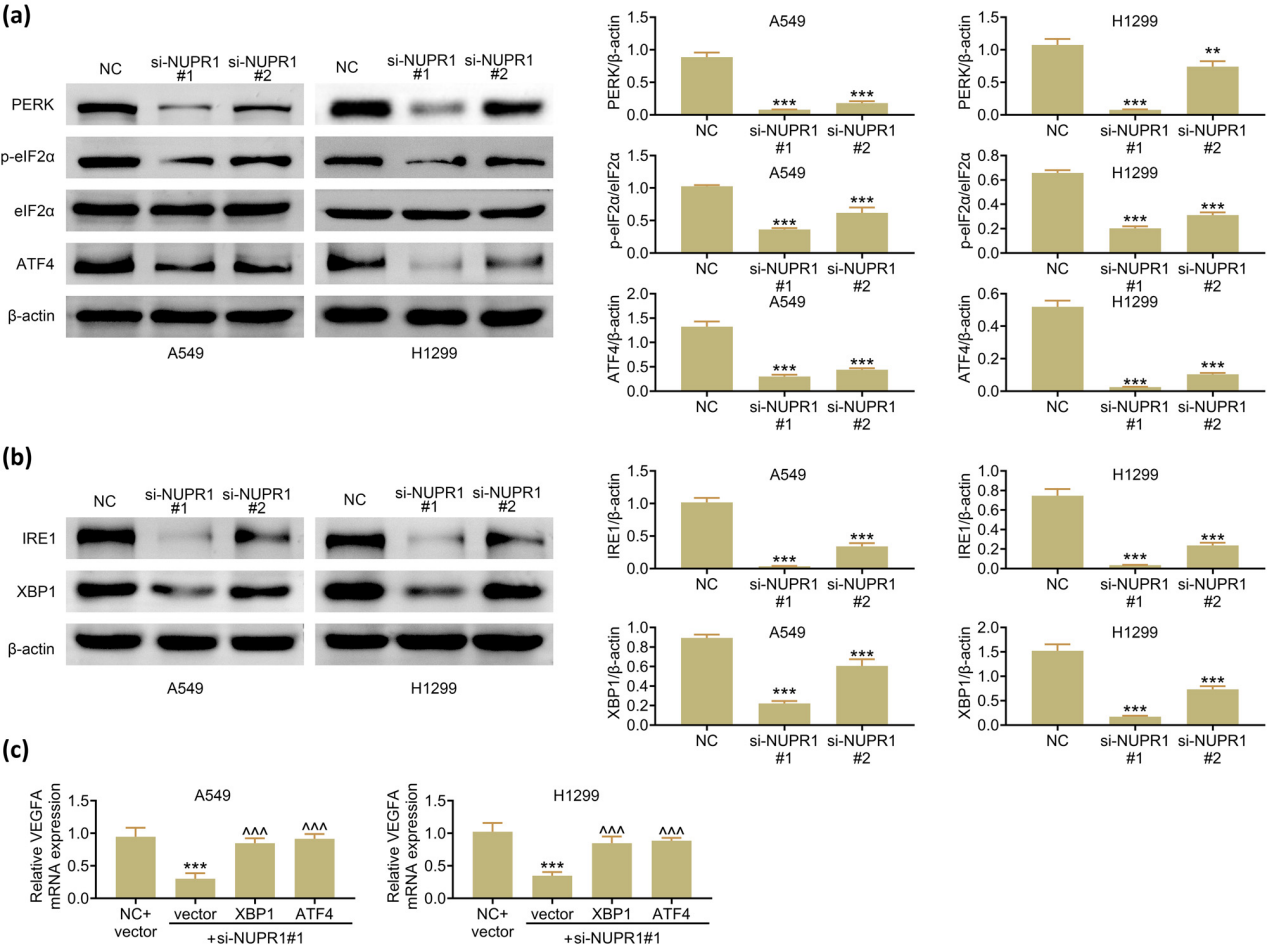
NUPR1 knockdown represses VEGFA expression through XBP1 and ATF4: (a) western blot analysis of PERK, p-eIF2α, eIF2α, and ATF4, and normalized with β-actin, (b) western blot analysis of IRE1 and XBP1 and normalized with β-actin, and (c) RT-qPCR analysis of VEGFA mRNA levels in A549 and H1299 cells. **p < 0.01, ***p < 0.001 vs the NC group. ^^^*p* < 0.001 vs the vector with si-NUPR1 group.

Chipurupalli et al. found that PERK–eIF2α–ATF4 signaling pathway confers a survival advantage to tumor cells under hypoxic environment [14]. Additionally, hypoxic activates the expression levels of XBP1 mRNA and protein, and the deficiency of XBP1 inhibits tumor growth [14]. Then, to clarify whether the mechanism of NUPR1 affected the reduction of VEGFA, and whether the effects may exert through acting on XBP1 and ATF4, the expression of PERK, p-eIF2α, eIF2α, ATF4, IRE1, and XBP1 in A549 and H1299 cells were detected by western blot. Data suggest that these XBP1 and ATF4 pathway-related molecules (Figure 4a and b) and VEGFA (Figure 4c) were dramatically decreased in NUPR1 knockdown group than in the control group. Furthermore, to determine the relationship between NUPR1 and XBP1 and ATF4 transcription factors, si-NUPR1 transfected cells supplied with XBP1 or ATF4 have upregulated the expression of VEGFA compared to si-NUPR1 with vector group (Figure 4c), which demonstrated that si-NUPR1 may repress the secretion of VEGFA by deregulating XBP1 and ATF4.

### 3.5 NUPR1 knockdown decreases angiogenesis by reducing VEGFA expression

**Figure 5:**
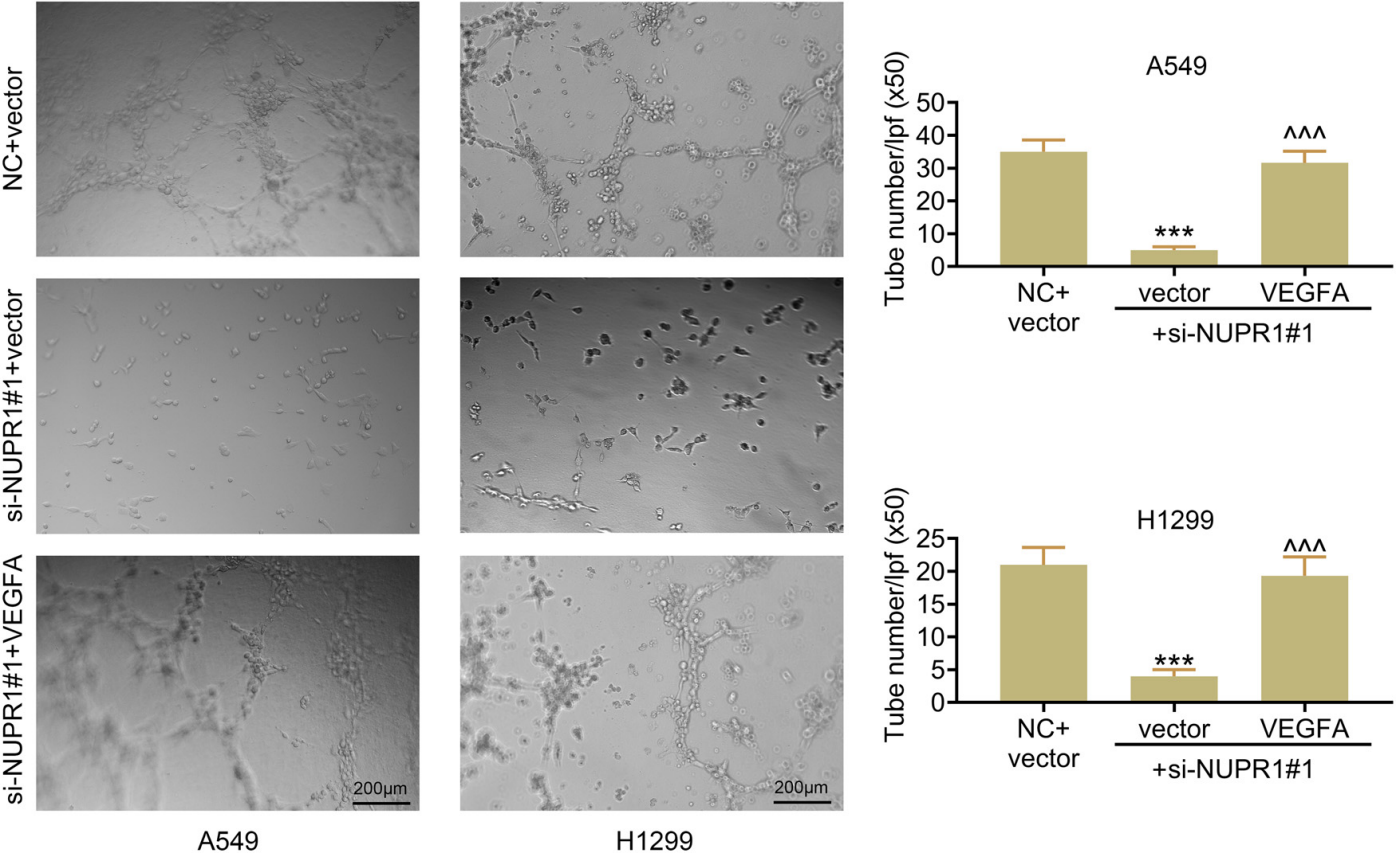
NUPR1 knockdown decreases angiogenesis by reducing VEGFA expression. Tube formation assays were performed to measure angiogenesis in HUVEC cells. ***p < 0.001 vs the NC group. ^^^*p* < 0.001 vs the vector with si-NUPR1 group.

VEGFA, a key angiogenic regulator, involves in the formation of the vascular network [13]. Here, HUVEC cells were seeded in the plate and cultured in A549 or H1299 cell medium, and measured angiogenesis through tube formation assay. We then found that NUPR1 knockdown significantly decreased the tube number in HUVEC cells, while cells in si-NUPR1 medium added with VEGFA stimulated the activities of angiogenesis than cells in si-NUPR1 (Figure 5), which suggests that the deletion of NUPR1 may decrease angiogenesis by reducing VEGFA expression.

## 4 Discussion

In this study, the knockdown of NUPR1 could inhibit angiogenesis in A549 and H1299 cells through IRE1/XBP1 and PERK/eIF2α/ATF4 signaling pathways. These findings first provide novel evidence for NUPR1 as a therapeutic target and its specific mechanism on lung cancer.

NUPR1, as a stress-induced protein, appears to be involved in a variety of stress-related functions [11] and can promote tumor growth and aggressiveness [9]. Previous studies have shown that NUPR1 affects the biological functions of tumor cells by regulating cell apoptosis and proliferation, and angiogenesis [12]. For example, NUPR1 alleviates apoptosis by promoting UPR in NSCLC [11]. NUPR1 controls the expression of genes critical to angiogenesis and tumor cell migration and invasion, facilitating the development and metastasis of metastatic breast cancer [15].

It is well established that angiogenesis and angiogenic factors contribute to several processes, which are important to cancer development and progression [3,16]. For instance, VEGF/VEGFR acts as a therapeutic target to inhibit metastasis and angiogenesis of NSCLC [3]. VEGFA, as the major pro-angiogenetic molecule, is induced by thyroid hormone, thereby accelerating angiogenesis and metastasis of liver cancer [12]. Vitexin, a bioactive flavonoid compound, inhibits the angiogenesis of cervical cancer through the VEGFA/VEGFR2 pathway, contributing to inhibition of tumor progression [16]. Similarly, our previous study also confirmed that NUPR1 knockdown inhibited the angiogenic activity of NSCLC by suppressing UPR-related factors (including XBP1 and others) and VEGFA activity [11]. In this study, NUPR1 was successfully silenced in lung cancer cell lines (A549 and H1299 cells) using si-RNA technology to investigate the effect of NUPR1 silencing on cell biological functions such as angiogenesis and migration in lung cancer. It was found that HUVEC cells cultured with cultures of si-NUPR1-transfected cancer cells showed a significant reduction in tube formation activity and inhibition of VEGFA expression. Further studies revealed that HUVEC tube formation activity was significantly improved by exogenous supplementation of VEGFA. These results suggest that si-NUPR1 may affect tumor progression by inhibiting the secretion of VEGFA to reduce angiogenesis.

In addition, it was shown that NUPR1 actively participates in the activation of the UPR, which may be responsible for the secretion of many pro-angiogenic factors, including VEGFA, XBP1, and ATF4 [17]. In this study, si-NUPR1 transfected cells were exogenously supplied with XBP1 or ATF4, which are downstream of NUPR1. These findings demonstrated that si-NUPR1 can inhibit VEGFA secretion by downregulating the activity of IRE1/XBP1 and PERK/eIF2α/ATF4 signaling pathways to reduce angiogenesis.

In conclusion, this study shows that si-RNA-mediated knockdown of NUPR1 significantly inhibited tubule formation activity in lung cancer and suppressed the cell migration activity, thereby suppressing tumor progression. The effect may be through the inhibition of the secretion of VEGFA, a pro-angiogenic factor, by downregulating the activity of IRE1/XBP1 and PERK/eIF2α/ATF4 signaling pathways. Further studies on the functional role of NUPR1 may lead to an improved understanding of the molecular mechanisms of lung cancer drugs, and inhibition of NUPR1 action contributing to the reduction of angiogenesis may be an effective strategy for the treatment of lung cancer.

## References

[1] Herbst RS, Heymach JV, Lippman SM. Lung cancer. N Engl J Med. 2008;359(13):1367–80. 10.1056/NEJMra0802714. PMC10662965 18815398

[2] Yu J, Peng W, Xue Y, Li Y, Yang L, Geng Y. FUBP1 promotes the proliferation of lung squamous carcinoma cells and regulates tumor immunity through PD-L1. Allergol Immunopathol. 2022;50(5):68–74. 10.15586/aei.v50i5.659 36086966

[3] Zhao Y, Guo S, Deng J, Shen J, Du F, Wu X, et al. VEGF/VEGFR-targeted therapy and immunotherapy in non-small cell lung cancer: targeting the tumor microenvironment. Int J Biol Sci. 2022;18(9):3845–58. 10.7150/ijbs.70958. PMC9254480 35813484

[4] Zhou H, Wang K, Wang M, Zhao W, Zhang C, Cai M, et al. ER-phagy in the occurrence and development of cancer. Biomedicines. 2022;10(3):707. 10.3390/biomedicines10030707. PMC8945671 35327508

[5] Chou CK, Liu W, Hong YJ, Dahms HU, Chiu CH, Chang WT, et al. Ethyl acetate extract of Scindapsus cf. hederaceus exerts the inhibitory bioactivity on human non-small cell lung cancer cells through modulating ER stress. Int J Mol Sci. 2018;19(7):1832. 10.3390/ijms19071832. PMC6073426 29933620

[6] Yoo YS, Han HG, Jeon YJ. Unfolded protein response of the endoplasmic reticulum in tumor progression and immunogenicity. Oxid Med Cell Longev. 2017;2017:2969271. 10.1155/2017/2969271. PMC5752989 29430279

[7] Wang S, Lou N, Luo R, Hao X, Liu Y, Wang L, et al. Role of chemokine-mediated angiogenesis in resistance towards crizotinib and its reversal by anlotinib in EML4-ALK positive NSCLC. J Transl Med. 2022;20(1):248. 10.1186/s12967-022-03451-2. PMC9153090 35642002

[8] Duan Q, Ni L, Wang P, Chen C, Yang L, Ma B, et al. Deregulation of XBP1 expression contributes to myocardial vascular endothelial growth factor-A expression and angiogenesis during cardiac hypertrophy in vivo. Aging Cell. 2016;15(4):625–33. 10.1111/acel.12460. PMC4933664 27133203

[9] Murphy A, Costa M. Nuclear protein 1 imparts oncogenic potential and chemotherapeutic resistance in cancer. Cancer Lett. 2020;494:132–41. 10.1016/j.canlet.2020.08.019. PMC7958295 32835767

[10] Guo X, Wang W, Hu J, Feng K, Pan Y, Zhang L, et al. Lentivirus-mediated RNAi knockdown of NUPR1 inhibits human nonsmall cell lung cancer growth in vitro and in vivo. Anat Rec (Hoboken). 2012;295(12):2114–21. 10.1002/ar.22571. 22961798

[11] Sun Y, Wang L, Deng C, Liu H, Guo Z, Chang Z, et al. Nuclear protein 1 promotes unfolded protein response during endoplasmic reticulum stress, and alleviates apoptosis induced by cisplatin in non-small cell lung cancer cells. Trop J Pharm Res. 2022;20:519–24. 10.4314/tjpr.v20i3.11.

[12] Chen CY, Wu SM, Lin YH, Chi HC, Lin SL, Yeh CT, et al. Induction of nuclear protein-1 by thyroid hormone enhances platelet-derived growth factor A mediated angiogenesis in liver cancer. Theranostics. 2019;9(8):2361–79. 10.7150/thno.29628. PMC6531305 31149049

[13] Pullamsetti SS, Banat GA, Schmall A, Szibor M, Pomagruk D, Hanze J, et al. Phosphodiesterase-4 promotes proliferation and angiogenesis of lung cancer by crosstalk with HIF. Oncogene. 2013;32(9):1121–34. 10.1038/onc.2012.136. 22525277

[14] Chipurupalli S, Kannan E, Tergaonkar V, D’Andrea R, Robinson N. Hypoxia induced ER stress response as an adaptive mechanism in cancer. Int J Mol Sci. 2019;20(3):749. 10.3390/ijms20030749. PMC6387291 30754624

[15] Hollern DP, Honeysett J, Cardiff RD, Andrechek ER. The E2F transcription factors regulate tumor development and metastasis in a mouse model of metastatic breast cancer. Mol Cell Biol. 2014;34(17):3229–43. 10.1128/MCB.00737-14. PMC4135561 24934442

[16] Wang Q, Zhang J, Ye J, Guo J. Vitexin exerts anti-tumor and anti-angiogensis effects on cervical cancer through VEGFA/VEGFR2 pathway. Eur J Gynaecol Oncol. 2022;43(4):86–91.

[17] Pereira ER, Liao N, Neale GA, Hendershot LM. Transcriptional and post-transcriptional regulation of proangiogenic factors by the unfolded protein response. PLoS One. 2010;5(9):e12521. 10.1371/journal.pone.0012521. PMC2932741 20824063

